# Rubiacordone A: A New Anthraquinone Glycoside from the Roots of *Rubia cordifolia*

**DOI:** 10.3390/molecules14010566

**Published:** 2009-01-23

**Authors:** Xiang Li, Zhi Liu, Yun Chen, Li-Juan Wang, Yi-Nan Zheng, Guang-Zhi Sun, Chang-Chun Ruan

**Affiliations:** 1 College of Chinese Medicinal Materials, Jilin Agricultural University, Changchun, 130118, P.R. China; E-mails: drxiang@hotmail.com (X. L.); ljwang@yahoo.com (L-J. W.); zhenyinan@tom.com (Y-N. Z.); 2 Institute of Agricultural Modernization, Jilin Agricultural University, Changchun, 130118, P.R. China; E-mails: jlndxdhs@126.com (Z. L.); gzsun1967@yahoo.com (G-Z. S.); 3 Research Centre for Medical and Structural Biology, School of Basic Medical Science, Wuhan University, Wuhan, 430071, P.R. China; E-mail: yunchen@whu.edu.cn (Y. C.)

**Keywords:** *Rubia cordifolia*, Rubiacordone A, Anthraquinone, Antimicrobial.

## Abstract

A new anthraquinone, Rubiacordone A (**1**) (6-acetoxy-1-hydroxy-2-methylanthraquinone-3-*O*-*α*-*l*-rhamnopyranoside), was isolated together with the known anthraquinone, 1-acetoxy-6-hydroxy-2-methylanthraquinone-3-*O*-[*α*-*l*-rhamnopyranosyl-(1→2)-*β*-*d*-glucopyranoside] (**2**), from the dried roots of *Rubia cordifolia*. Their structures were elucidated on the basis of extensive 1D and 2D-NMR, as well as HRESI-MS spectroscopic analysis. Metabolites **1** and **2** showed considerable antimicrobial activity against Gram-positive bacteria.

## Introduction

*Rubia cordifolia* Linn (*Rubiaceae*) is a slender, branched, climbing plant, with very long cylindrical roots, widely distributed in China, India and tropical Australia [1]. In the traditional Chinese system of medicine, *Rubia cordifolia* is used for the treatment of vertigo, insomnia, rheumatism, tuberculosis, hematemesis, menstrual disorders and contusions. The plant was recently reported to possess antimicrobial activity [2,3] and previous phytochemical examinations have shown that it produces triterpenoids, anthraquinones, cyclopeptides and phenolics [4,5,6,7,8,9,10,11,12]. During our investigations on plants used for antimicrobial purposes in Chinese medicine, a 70% methanolic extract of the dried roots of *Rubia cordifolia* showed considerable activity against Gram-positive and Gram-negative bacteria. Purification of the bioactive extract results in the isolation and identification of a novel anthraquinone, designated with the common named Rubiacordone A (**1**), and the previously reported anthraquinone **2**, both of which showed antimicrobial activity against Gram-positive bacteria when tested using the disc-diffusion method.

## Results and Discussion

### Characterization of compound **1**

**Figure 1:**
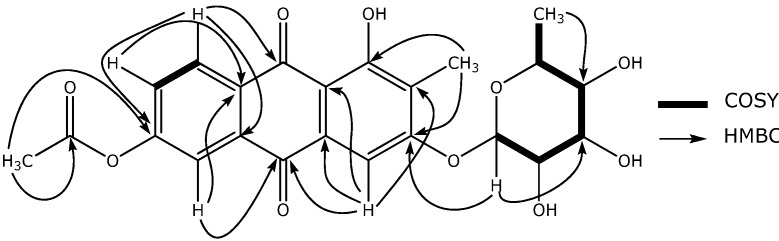
HMBC and COSY correlations of compound **1**.

Anthraquinone **1** was isolated as an orange-colored amorphous powder, and its molecular formula was determined as C_23_H_22_O_10_ on the basis of its HR-ESI-MS (*m/z* 459.1303 [M+H]^+^, *calcd.* 459.1291) and NMR data (Table 1). While, the IR spectra of **1** exhibited absorption bands at 3,438, 1,739 and 1,630 cm^-1^, indicating the existence of hydroxyl, acetyl and ketone functionalities in the structure, the UV spectra showed typical anthraquinone maxima values at 269, 305, 410 nm. Analysis of the ^13^C-NMR spectrum and the DEPT experiment, allowed the identification of three methyl groups, nine methines and nine quaternary carbons. The chemical shifts of the protons and carbons in the phenolic region of **1** (Table 1) were similar to those of the previously reported 1-acetoxy-6-hydroxy-2-methylanthraquinone-3-*O*-[*α*-*l*-rhamnopyranosyl-(1→2)-*β*-*d*-glucopyranoside (**2**), an anthraquinone also isolated from *Rubia cordifolia* [8]. The main differences between the two metabolites concerned the location of the acetyl group and the nature of the sugar moiety; with the acetyl group located at C-1 in **2** and to C-6 in **1**, and the sugar moiety being *α*-*l*-rhamnopyranoside in the novel anthraquinone (**1**), instead of the *α*-*l*-rhamnopyranosyl-(1→2)-*β*-*d*-glucopyranoside present in the known anthraquninone **2**. This was supported by the ^13^C-NMR data of **1**, which showed only one set of “sugar” signals at *δ*_C_ 103.3 (d, C-1'), *δ*_C_ 71.7 (d, C-2'), *δ*_C_ 72.9 (d, C-3'), *δ*_C_ 72.0 (d, C-4'), *δ*_C_ 73.2 (d, C-5'), and *δ*_C_ 18.6 (q, C-6'''). The location of the sugar moiety at C-3 was established taking into account the correlation observed between H-1’ (*δ* 5.48) and C-3 (*δ* 156.8) in the HMBC experiment of **1** (Figure 1). The sugar moiety was identified as rhamnose when the acid hydrolysis of **1** produced a component having the same *R_f_* value on TLC as that of an authentic sample of rhamnose. The coupling constant between the H1' and H2' (1.5 Hz) protons in the ^1^H-NMR of **1** indicated an *α*-rhamnose; the absolute configuration of the *α*-rhamnose was further determined to be *α*-*l*-rhamnose by chiral GC analysis. Finally, the location of the acetyl group was established on the basis of the HMBC correlation between the acetyl’s methyl group (*δ* 2.10) and the C-6 carbon (158.7) (Figure 1). On the basis of this data, metabolite **1** was identified as 6-acetoxy-1-hydroxy-2-methylanthraquinone-3-*O*-*α*-*L*-rhamnopyranoside, to which we have given the common name Rubiacordone A. 

**Table 1:** ^1^H- (500 Hz) and ^13^C- (125 Hz) NMR data for Rubiacordone A (**1**)^a^.

Position	^1^H-NMR	*J* (Hz)	^13^C-NMR	DEPT	COSY	HMBC
**1**	-	-	162.0	C	-	-
**2**	-	-	123.4	C	-	-
**3**	-	-	156.8	C	-	-
**4**	7.35, *s*	-	104.6	CH	-	C-2, C-4a, C-9a, C-10
**4a**	-	-	132.7	C	-	-
**5**	7.45, *d*	2.4	113.1	CH	H-7	C-7, C-8a, C-10
**6**	-	-	158.7	C	-	-
**7**	7.21, *dd*	8.0, 2.4	120.5	CH	H-5, H-8	C-5, C-8a
**8**	7.95, *d*	8.0	129.1	CH	H-7	C-6, C-9, C-10a
**8a**	-	-	124.5	C	-	-
**9**	-	-	N.D. ^c^	-	-	-
**9a**	-	-	108.6	C	-	-
**10**	-	-	N.D. ^c^	-	-	-
**10a**	-	-	131.7	C	-	-
**Ac-C=O**	-	-	169.2	C	-	-
**Ac-Me**	2.10, *s*	-	24.3	CH_3_	-	Ac-C=O, C-6
**2-CH_3_**	1.05, *s*	-	8.7	CH_3_	-	C-1, C-2, C-3
**Rha**						
**1’**	5.48, *d*	1.5	103.3	CH	H-2’	C-5’, C-3’
**2’**	4.22, *dd*	1.5, 3.4	71.7	CH	H-1’, H-3’	C-3’, C-4’
**3’**	3.79, *dd*	3.4, 10.0	72.9	CH	H-2’, H-4’	C-1’, C-5’
**4’**	3.36, *dd*	9.5, 10.0	72.0	CH	H-3’, H-5’	C-2’, C-5’ C-6’
**5’**	3.45, m	-	73.2	CH	H-4’, H-6’	C-1’, C-3’
**6’**	0.88, *d*	6.0	18.6	CH_3_	H-5’	C-4’, C-5’

^a^ Measured in DMSO-*d*_6_; chemical shifts are expressed in ppm.

### Bioactivity Results

The antimicrobial activities of 20 μg of the isolated metabolites (in 50 μL DMSO) against five Gram-positive bacteria (*S. aureus*, *B. subtilis*, *S. epidermidis*, *S. faecalis*, and *B. cereus*) and four Gram-negative bacteria (*V. parahaemolyticus*, *P. aeruginosa*, *S. typhimurium*, and *E. coli*) were determined using the agar-disc diffusion method [13]. Metabolites **1** and **2** showed a similar pattern on antimicrobial activity, with both of them showing activity towards the Gram-positive bacteria *B. subtilis*, *S. faecalis* and *B. cereus* (Table 2).

**Table 2:** Antimicrobial activities of 70% methanolic extract and isolated compounds ^a^.

		Inhibition zone diameter (mm)			
Microorganism	CE^b^	1	2	Amracin
	200 *μ*g/disc	20 *μ*g/disc	20 *μ*g/disc	10 *μ*g/disc
*S. aureus*	6 ± 0.5	13 ± 0.3	19 ± 0.6	32 ± 0.5
*B. subtilis*	16 ± 0.6	26 ± 0.5	27 ± 0.3	41 ± 0.3
*S. epidermidis*	7 ± 0.3	14 ± 0.4	11 ± 0.5	36 ± 0.6
*S. faecalis*	14 ± 0.6	29 ± 0.2	22 ± 0.5	35 ± 0.5
*B. cereus*	14 ± 0.3	24 ± 0.6	26 ± 0.5	35 ± 0.5
*V. parahaemolyticus*	10 ± 0.6	8 ± 0.5	11 ± 0.3	24 ± 0.6
*P. aeruginosa*	7 ± 0.5	9 ± 0.5	8 ± 0.6	21 ± 0.4
*S. typhimurium*	6 ± 0.4	12 ± 0.2	8 ± 0.3	32 ± 0.5
*E. coli*	13 ± 0.4	10 ± 0.4	11 ± 0.5	28 ± 0.4

^a^ Mean value ± SD, *n* = 3 (the zone of inhibition including disc of 6 mm in diameter). ^b^ CE: 70% MeOH Crude Extract.

## Conclusions

In summary, we have isolated a novel anthraquinone glycoside, rubiacordone A (**1**), from the dried roots of *Rubia cordifolia* together with the known compound **2**. The isolated compounds **1** and **2** were tested for their antimicrobial activities against Gram-positive and Gram-negative bacteria by the disc diffusion method. Both compounds were found to have antimicrobial effects against the Gram-positive bacteria *B. subtilis*, *S. faecalis* and *B. cereus*.

## Experimental

### General

The ^1^H- and ^13^C-NMR spectra were measured on a Bruker Avance DRX 500 NMR spectrometer (Bruker Bio-Spin, Rheinstetten, Germany). Chemical shifts (*δ*) are expressed in parts per million (ppm) and coupling constants (*J*) in Hertz (Hz). The ESIMS spectra were recorded on a triple quadrupole mass spectrometer Quattro (VG Biotech, Altrincham, England) and the HRESI-MS spectra on a Bruker FT-ICRMS spectrometer. IR spectrum was recorded on a Thermo Nicolet Nexus 470 FT-IR spectrometer. Gel permeation column chromatography purifications were carried out using Sephadex LH-20 (Pharmacia); HPLC analyses were performed with an Agilent 1100 instrument using a Zorbax C_18_ column (150 x 25 mm, Phenomenex, Torrance, CA). UV absorption data (λ_280_) were obtained with an Agilent Chemstation Ver 8.01. All solvents used in this study were HPLC grade, purchased from Yu-dong Biotech. Co. Ltd., Shanghai, P.R. China. Other chemicals were purchased from the Chinese Chemical Group, Beijing, P.R. China. 

### Extraction and isolation

The dry roots of *Rubia cordifolia* were purchased from Hu-jiao Chinese Medicinal Material Company, An-Hui, China and identified by one of the authors, Prof. Yi-Nan Zheng. A voucher specimen (Qian-Cao-001) was deposited in the Laboratory of Medicinal Chemistry, JLAU. The powdered roots (1.1 kg) of roots of *Rubia cordifolia* were extracted three times for 48 h with 4 L of 70% MeOH at room temperature; the extract was concentrated to give a dark brown residue (273 g), which was re-dissolved in 50% MeOH and subjected to gel permeation column chromatography, eluting with MeOH: H_2_O: AcOH (10:10:1), to yield six fractions (F_A_ – F_F_). The third fraction (F_C_) was purified by semipreparative HPLC (gradient elution of 5% aqueous MeOH to 100% MeOH) to yield metabolite **1** (3 mg). Purification of the fourth fraction (F_D_) by silica gel column chromatography, eluting with CHCl_3_-MeOH-H_2_O (4:4:1), resulted in five new fractions (F_D-1_ to F_D-5_). F_D-2_ was purified by semipreparative HPLC, using the same elution conditions as mentioned above, to afford metabolite **2** (81 mg).

### Acid hydrolysis of compound **1**

Anthraquinone **1** (2.0 mg) was refluxed with 6 N HCl (5 mL) at 100 °C for 2 h. The reaction mixture was extracted with CH_2_Cl_2_ to obtain the corresponding aglycone; the aqueous layer was neutralized with Na_2_CO_3_, filtered, and dried under vacuum. The resulting residue was re-dissolved in H_2_O for sugar analysis on TLC, eluting with *n*-BuOH–HOAc–H_2_O (6:1:2). The sample spots were detected by spraying aniline hydrogen phthalate reagent (100 mL *n*-BuOH saturated by H_2_O, 0.96 g aniline and 1.66 g phthalic acid) and heating. Glucose, xylose and rhamnose were used as authentic standards. 

### Absolute configuration of rhamnose

The absolute configuration of rhamnose was determined by chiral GC analysis using a SatoChrom GC and a 0.25 mm x 25 m Hydrodexb-6-TBDM chiral capillary column (Macherey-Nagel, Germany), and *α*-*L*-rhamnose as an authentic GC standard. To prepare the sample for analysis, the aqueous residues mentioned above was re-suspended in dichloromethane (1 mL) and treated with trifluoroacetic anhydride (50 µL). The mixture was allowed to react at room temperature for 16h and then dried under a stream of nitrogen at room temperature. The sugar derivative was analyzed using the following temperature program: inlet temperature was set at 240 °C, with hydrogen carrier gas and a 1/20 split, using nitrogen makeup gas. Column temperatures starting at 120 °C, climbing to 220 °C at 50 °C min^–1^ and maintaining the temperature for 12 min.

*Rubiacordone A* (**1**). Obtained as an orange-colored amorphous powder, mp. 251 – 252 °C; UV (MeOH) λ_max_: 269, 305, 410 nm; IR *ν* (KBr) cm^-1^: 3,438 (OH), 1,739 (C=O), 1,630 (C=O); HRESI-MS [+]: *m/z* = 459.1303 [M+H]^+^ (*calcd*. for C_23_H_23_O_10_, 459.1291); ^1^H and ^13^C-NMR data, see Table 1.

### Antimicrobial assay

The antimicrobial activity of samples was determined using the agar-disc diffusion method [13] with a slight modification. Five Gram-positive bacteria (*S. aureus*, *B. subtilis*, *S. epidermidis*, *S. faecalis*, and *B. cereus*) and four Gram-negative bacteria (*V. parahaemolyticus*, *P. aeruginosa*, *S. typhimurium*, and *E. coli*) originally isolated from various sources were kindly provided and identified by Professor Zhao-yang He (Animal Science Department, Jilin Agricultural University). The nutrient agar plates were prepared with 25 mL nutrient broth containing a 2% agar (Difco). Five milliliters of top nutrient agar (1% agar, 45 °C) containing a 10% inoculums of microbial strains with an OD_600nm_ = 1 was poured and uniformly spread on each agar plate. After the solidification of the top agar, the sterile paper discs were placed onto the plate, and the sample solution (20 μg/50 μL DMSO) was then applied to each paper disc. Each plate was incubated at 37 °C for 24 h and the growth inhibition zones were measured. Amracin (10 μg /disc) (Sigma-Aldrich) was used as positive control.
